# Supplementary material for: Assessing the global risk of typhoid outbreaks caused by extensively drug resistant Salmonella Typhi
Source: Nat Commun. 2023 Oct 16;14:6502. doi: 10.1038/s41467-023-42353-9 (PMC10579367; doi:10.1038/s41467-023-42353-9)
Supplement: Supplementary file 1 — Supplementary Information [file 41467_2023_42353_MOESM1_ESM.pdf]

Supplementary Information

Supplementary Table 1. Countries with known XDR *S*. Typhi importations

	Country	World Bank Region (Income Group)	Air Travelers from Pakistan, 2019 (Percentile)	Median Estimated Typhoid Incidence per 100,000 Person-Years (Burden Group)	Known XDR Typhoid Importations
1	United Arab Emirates	Middle East & North Africa (High)	1,672,858 (99)	0 (None)	1 case diagnosed following travel from Pakistan in 2019[30]
2	United Kingdom	Europe & Central Asia (High)	719,099 (98.5)	0 (None)	64 cases diagnosed following travel from Pakistan as of March 2020[27]
3	United States	North America (High)	349,938 (98)	0 (None)	67 cases diagnosed following travel from Pakistan as of March 2021[32]
4	Oman	Middle East & North Africa (High)	240,448 (97.5)	0 (None)	3 cases diagnosed following travel from Pakistan in 2019[31]
5	China	East Asia & Pacific (Upper-Middle)	204,851 (97)	22.3 (Low)	Local outbreak[40]
6	Qatar	Middle East & North Africa (High)	154,783 (96.5)	0 (None)	1 case diagnosed following travel from Pakistan in 2019[29]
7	Canada	North America (High)	127,738 (95.5)	0 (None)	10 cases diagnosed following travel from Pakistan as of March 2021[33]
8	Italy	Europe & Central Asia (High)	104,829 (94)	0 (None)	1 case diagnosed following travel from Pakistan in 2019[35]
9	Spain	Europe & Central Asia (High)	68,124 (92)	0 (None)	1 case diagnosed following travel from Pakistan in 2018[34]
10	Australia	East Asia & Pacific (High)	64,116 (91)	0 (None)	4 cases diagnosed following travel from Pakistan in 2019 and 2020[37,38]
11	Denmark	Europe & Central Asia (High)	26,526 (88.5)	0 (None)	1 case diagnosed following travel from Pakistan in 2019[36]
12	Hong Kong	East Asia & Pacific (High)	25,761 (87.5)	0 (None)	1 case in 2022 with no recent travel history[41]
13	Norway	Europe & Central Asia (High)	23,885 (86.5)	0 (None)	1 case diagnosed following travel from Pakistan in 2019[33]
14	Ireland	Europe & Central Asia (High)	10,451 (80)	0 (None)	3 cases diagnosed following travel from Pakistan as of October 2019[52]
15	India	South Asia (Lower-Middle)	4,907 (75)	438.9 (High)	1 case diagnosed in the UK following travel from India in 2019[27]
16	Taiwan	East Asia & Pacific (High)	1,057 (60)	0 (None)	1 case diagnosed following travel from Pakistan in 2019[39]

Supplementary Table 2. Countries receiving the most air passengers from Pakistan in 2019

	Country	World Bank Region (Income Group)	Air Travelers from Pakistan, 2019	Median Estimated Typhoid Incidence per 100,000 Person-Years (Burden Group)	Known XDR Typhoid Importations
1	Saudi Arabia	Middle East & North Africa (High)	2,636,806	0 (None)	None
2	United Arab Emirates	Middle East & North Africa (High)	1,672,858	0 (None)	1 case diagnosed following travel from Pakistan in 2019[30]
3	United Kingdom	Europe & Central Asia (High)	719,099	0 (None)	64 cases diagnosed following travel from Pakistan as of March 2020[27]
4	United States	North America (High)	349,938	0 (None)	67 cases diagnosed following travel from Pakistan as of March 2021[32]
5	Oman	Middle East & North Africa (High)	240,448	0 (None)	3 cases diagnosed following travel from Pakistan in 2019[31]
6	China	East Asia & Pacific (Upper-Middle)	204,851	22.3 (Low)	Local outbreak[38]
7	Qatar	Middle East & North Africa (High)	154,783	0 (None)	1 case diagnosed following travel from Pakistan in 2019[29]
8	Turkey	Europe & Central Asia (Upper-Middle)	132,378	12.9 (Low)	None
9	Canada	North America (High)	127,738	0 (None)	10 cases diagnosed following travel from Pakistan as of March 2021[33]
10	Malaysia	East Asia & Pacific (Upper-Middle)	124,384	128.6 (High)	None

Supplementary Table 3. High and very high typhoid burden countries receiving the most air passengers from Pakistan in 2019

	Country	World Bank Region (Income Group)	Air Travelers from Pakistan, 2019	Median Estimated Typhoid Incidence per 100,000 Person-Years (Burden Group)	Known XDR Typhoid Importations
1	Malaysia	East Asia & Pacific (Upper-Middle)	124,384	128.6 (High)	None
2	Thailand	East Asia & Pacific (Upper-Middle)	93,396	199.5 (High)	None
3	Sri Lanka	South Asia (Lower-Middle)	26,267	152.7 (High)	None
4	Afghanistan	South Asia (Low)	19,417	744.3 (Very High)	None
5	Indonesia	East Asia & Pacific (Lower-Middle)	11,489	140.2 (High)	None
6	Kenya	Sub-Saharan Africa (Lower-Middle)	8,304	209.9 (High)	None
7	Philippines	East Asia & Pacific (Lower-Middle)	5,939	310.6 (High)	None
8	Bangladesh	South Asia (Lower-Middle)	5,568	545.1 (Very High)	None
9	India	South Asia (Lower-Middle)	4,907	438.9 (High)	1 case diagnosed in the UK following travel from India in 2019[27]
10	Uganda	Sub-Saharan Africa (Low)	3,873	146.1 (High)	None

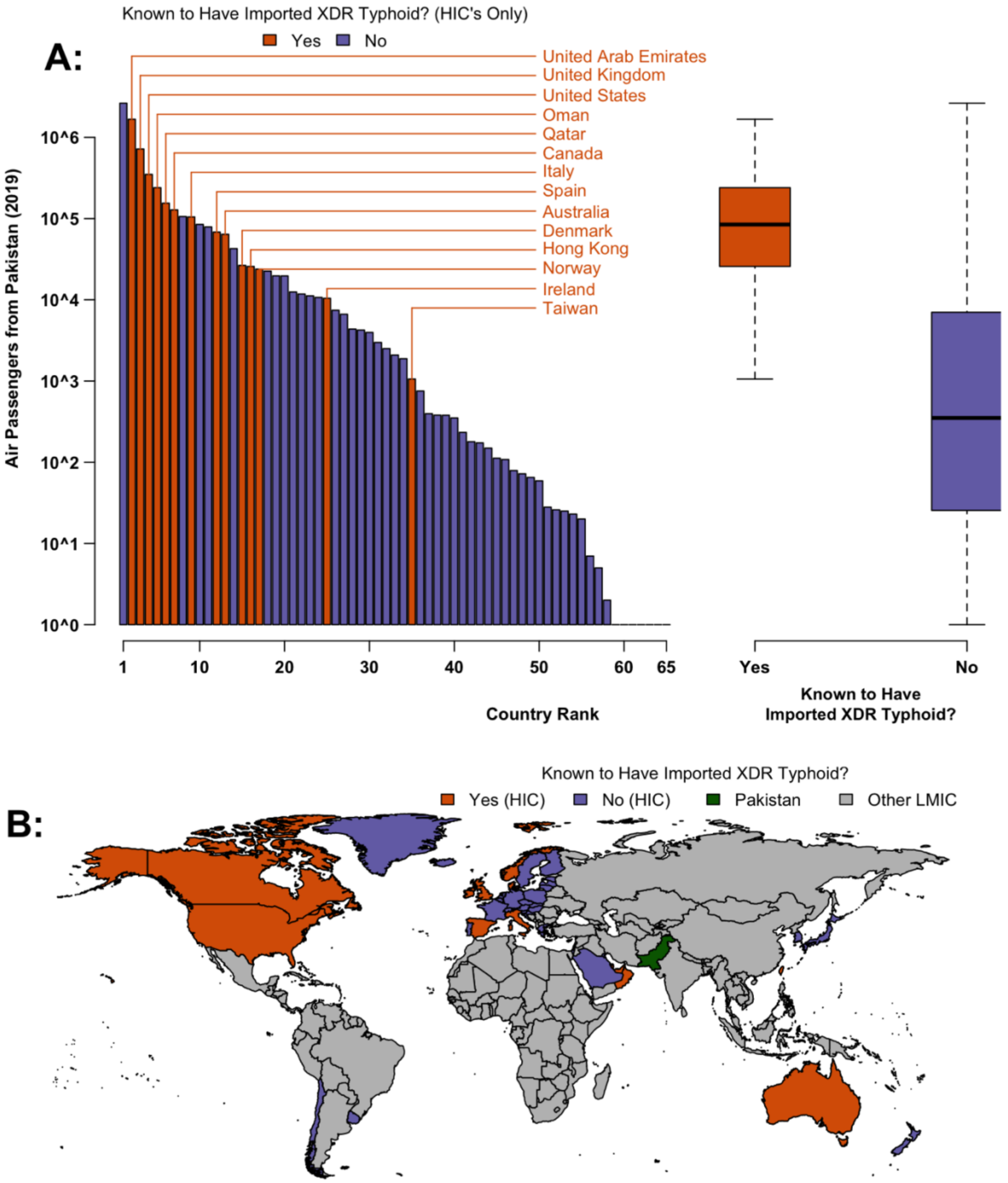

Supplementary Figure 1. Air Travel from Pakistan to high-income countries with and without known XDR *Salmonella* Typhi importations. (A) The barplot (left) and boxplot (right) show the volume of 2019 air travel from Pakistan to high-income countries (HIC) that are (orange, n = 14 countries) and are not (purple, n = 51 countries) known to have imported XDR *S.* Typhi cases to date. The number of travelers has been log10-transformed for interpretability. (B) Map of high-income countries by XDR *S*. Typhi importation status. Map data from the Natural Earth project. Source data is provided as a Source Data file.

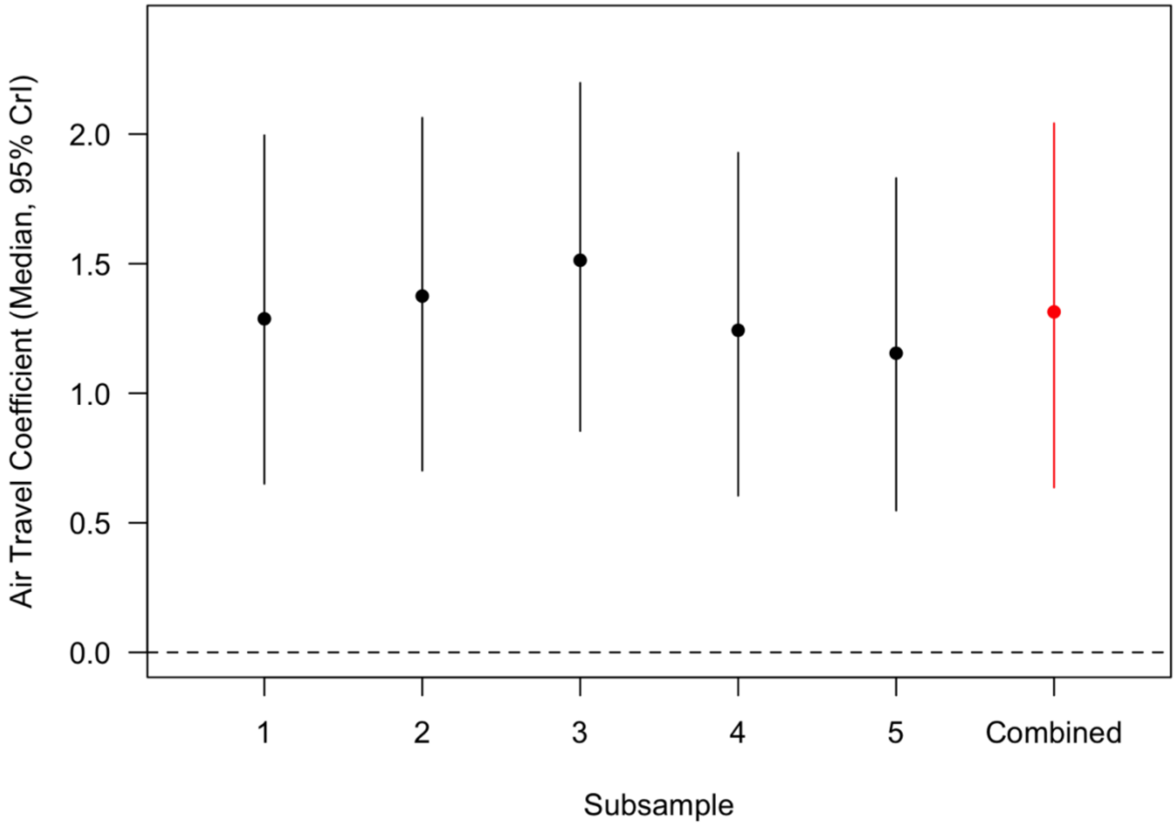

Supplementary Figure 2. Association Between Air Travel Volume and the Rate of Between-Country Movement for the H58 Haplotype of *S.* Typhi.
Positive coefficient values indicate a higher rate of H58 movement between countries with a greater exchange of air travelers. The combined estimate (red) is similar to those from each of the 5 individual subsamples of H58 sequences. Coefficient values were estimated as part of the BEAST phylogeographic analysis. Source data is provided as a Source Data file.

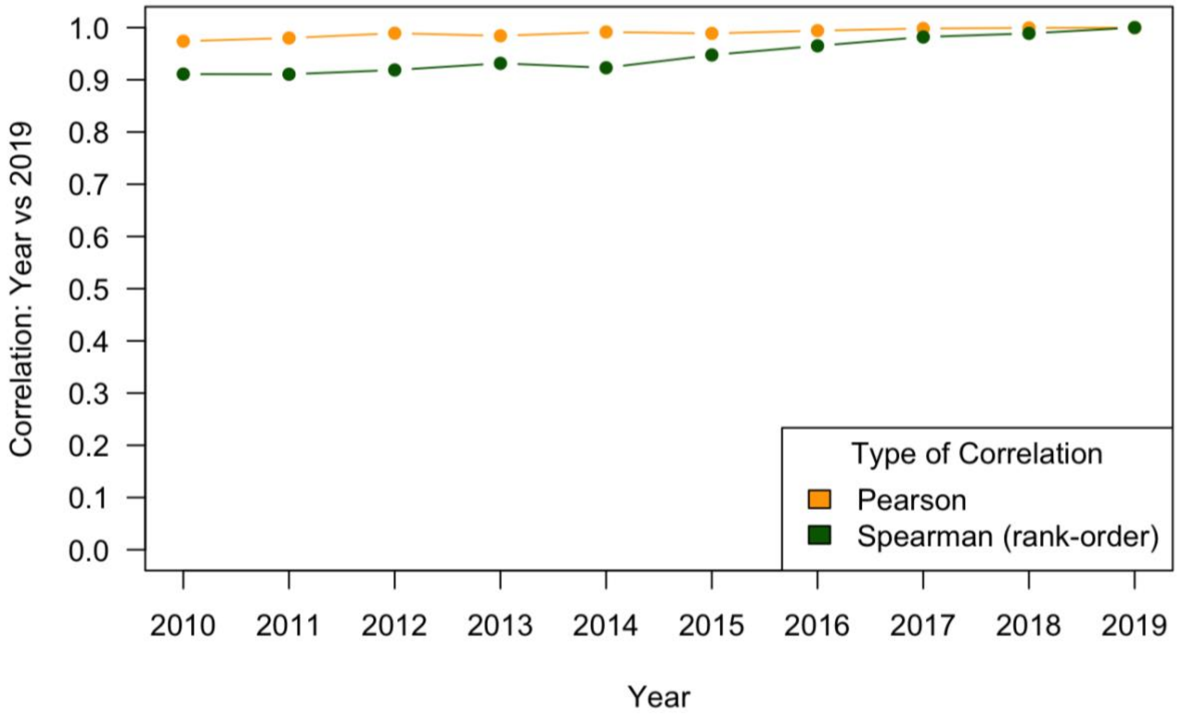

Supplementary Figure 3. Correlation between country-level air travel volume from Pakistan in 2019 versus previous years (2010-2018).
Source data is provided as a Source Data file.

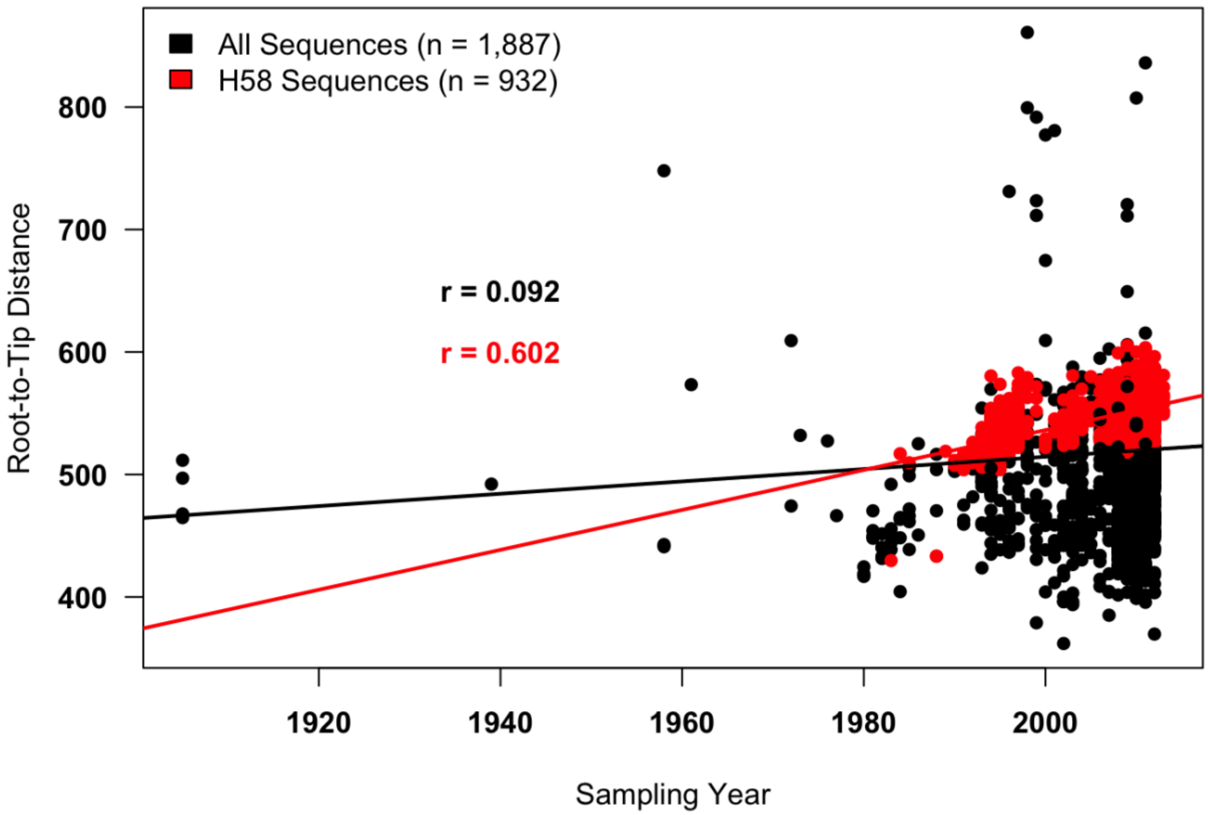

Supplementary Figure 4. Temporal Analysis of *Salmonella* Typhi Sequences
Each point corresponds to a specific *S.* Typhi sequence, with H58 sequences colored red, and all others colored black. Root-to-tip distances extracted from the maximum likelihood tree are plotted against the year of sampling. The strength of the temporal signal was relatively weak across all isolates (r = 0.092), but much stronger for the H58 sequences (r = 0.602). Source data is provided as a Source Data file.

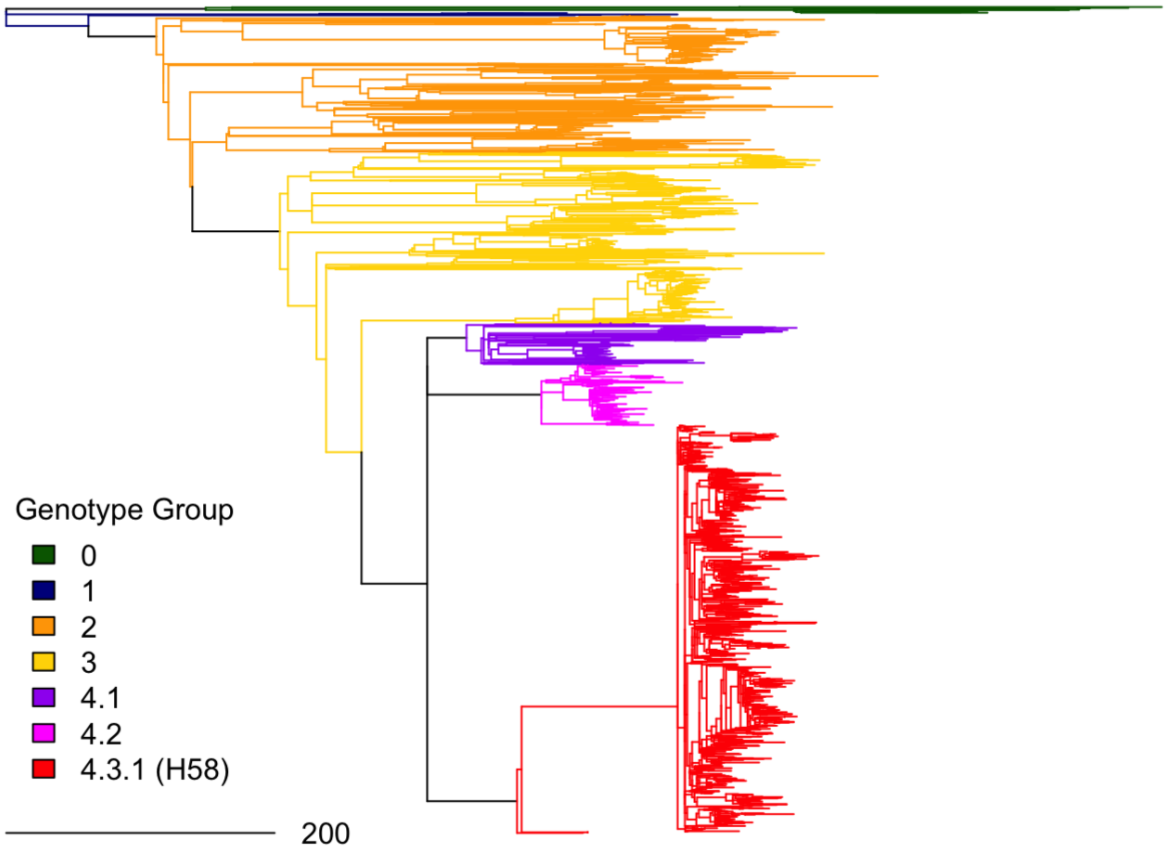

Supplementary Figure 5. Maximum Likelihood Phylogeny of *Salmonella* Typhi Sequences. Each genotype group contains the indicated genotype and all descendent sublineages. For instance, genotype group 4.3.1 includes genotype 4.3.1 as well as 4.3.1.1, 4.3.1.2, etc. The H58 haplotype (genotype group 4.3.1, colored red) forms a distinct monophyletic cluster.
