# Supplementary material for: Assessing the global risk of typhoid outbreaks caused by extensively drug resistant Salmonella Typhi
Source: Nat Commun. 2023 Oct 16;14:6502. doi: 10.1038/s41467-023-42353-9 (PMC10579367; doi:10.1038/s41467-023-42353-9)
Supplement: Supplementary file 3 — Description of Additional Supplementary Files [file 41467_2023_42353_MOESM3_ESM.pdf]

Description of Additional Supplementary Files

Supplementary Data 1: Number of H58 and non-H58 S. Typhi sequences by country
