# Supplementary material for: Assessing the global risk of typhoid outbreaks caused by extensively drug resistant Salmonella Typhi
Source: Nat Commun. 2023 Oct 16;14:6502. doi: 10.1038/s41467-023-42353-9 (PMC10579367; doi:10.1038/s41467-023-42353-9)
Supplement: Supplementary file 5 — Reporting Summary [file 41467_2023_42353_MOESM5_ESM.pdf]

nature portfolio

Corresponding author(s): Joseph Walker

Last updated by author(s): Sep 20, 2023

Reporting Summary

Nature Portfolio wishes to improve the reproducibility of the work that we publish. This form provides structure for consistency and transparency in reporting. For further information on Nature Portfolio policies, see our Editorial Policies and the Editorial Policy Checklist.

Statistics

For all statistical analyses, confirm that the following items are present in the figure legend, table legend, main text, or Methods section.

n/a	Confirmed	
☐	☒	The exact sample size (*n*) for each experimental group/condition, given as a discrete number and unit of measurement
☒	☐	A statement on whether measurements were taken from distinct samples or whether the same sample was measured repeatedly
☐	☒	The statistical test(s) used AND whether they are one- or two-sided<br>*Only common tests should be described solely by name; describe more complex techniques in the Methods section.*
☐	☒	A description of all covariates tested
☒	☐	A description of any assumptions or corrections, such as tests of normality and adjustment for multiple comparisons
☐	☒	A full description of the statistical parameters including central tendency (e.g. means) or other basic estimates (e.g. regression coefficient) AND variation (e.g. standard deviation) or associated estimates of uncertainty (e.g. confidence intervals)
☒	☐	For null hypothesis testing, the test statistic (e.g. *F*, *t*, *r*) with confidence intervals, effect sizes, degrees of freedom and *P* value noted<br>*Give P values as exact values whenever suitable.*
☐	☒	For Bayesian analysis, information on the choice of priors and Markov chain Monte Carlo settings
☒	☐	For hierarchical and complex designs, identification of the appropriate level for tests and full reporting of outcomes
☐	☒	Estimates of effect sizes (e.g. Cohen's *d*, Pearson's *r*), indicating how they were calculated

Our web collection on statistics for biologists contains articles on many of the points above.

Software and code

Policy information about availability of computer code

Data collection	No software was used.
Data analysis	Data analysis was performed using R (v3.6.3), RStudio (v1.4.1717), the ape (v5.6-1) and TreeTools (v1.8.0) R packages, Beast (v1.10.4), Tracer (v1.7.2), LogCombiner (v1.10.4), BEAUti (v1.10.4), snippy (v4.0.2), and gubbins (v2.3.4). All code needed to replicate this analysis can be accessed at github.com/joewalker127/XDR_Typhoid.

For manuscripts utilizing custom algorithms or software that are central to the research but not yet described in published literature, software must be made available to editors and reviewers. We strongly encourage code deposition in a community repository (e.g. GitHub). See the Nature Portfolio guidelines for submitting code & software for further information.

Data

Policy information about availability of data

All manuscripts must include a data availability statement. This statement should provide the following information, where applicable:

- Accession codes, unique identifiers, or web links for publicly available datasets
- A description of any restrictions on data availability
- For clinical datasets or third party data, please ensure that the statement adheres to our policy

The full genome sequences used in our phylogeographic analysis and corresponding metadata have been deposited in the pathogen.watch database at https://

pathogen.watch/collection/vlnn0nzwjfr0-walker-et-al-2023. All other non-genomic data needed to replicate this analysis can be accessed at github.com/joewalker127/XDR_Typhoid. Source data are provided with this paper.

Research involving human participants, their data, or biological material

Policy information about studies with human participants or human data. See also policy information about sex, gender (identity/presentation), and sexual orientation and race, ethnicity and racism.

Reporting on sex and gender	Our study does not involve human participants, data, or biological material. As such, this field is not applicable.
Reporting on race, ethnicity, or other socially relevant groupings	Not applicable
Population characteristics	Not applicable
Recruitment	Not applicable
Ethics oversight	Not applicable

Note that full information on the approval of the study protocol must also be provided in the manuscript.

Field-specific reporting

Please select the one below that is the best fit for your research. If you are not sure, read the appropriate sections before making your selection.

☐ Life sciences ☐ Behavioural & social sciences ☒ Ecological, evolutionary & environmental sciences

For a reference copy of the document with all sections, see nature.com/documents/nr-reporting-summary-flat.pdf

Ecological, evolutionary & environmental sciences study design

All studies must disclose on these points even when the disclosure is negative.

Study description	We fit a series of bayesian phylogeographic models to evaluate the impact of air travel on the global emergence and dispersal of H58 S. typhi.
Research sample	We utilized an existing dataset of 1,804 S. typhi consensus genomes (n=849 from the H58 haplotype) which were previously analyzed by Wong et al. in a 2015 Nature Genetics paper. This dataset was selected because it covers a range of countries over multiple decades. We supplemented this dataset with 83 early H58 genomes analyzed by Carey et al. The resulting sample of genomes is intended to represent the expanding global population of the H58 S. typhi organism following its emergence in South Asia in the 1980's.
Sampling strategy	Preexisting genomic data was included on the basis of availability. We then randomly selected a maximum of 16 H58 genomes from each country prior to phylogeographic model fitting, to avoid over- or under-representation of any individual country.
Data collection	We did not conduct any experiments as part of this study, only analyses of preexisting genomic data. This data and corresponding metadata (particularly the year and country of sampling, as specified during submission) was accessed from the pathogen.watch repository at https://pathogen.watch/genomes/all?collection=lxwpz49fpxp1-carey-et-al-2022 and https://pathogen.watch/genomes/all?collection=4wlhd6xzek8s-wong-et-al-2016.
Timing and spatial scale	Genomic data was available and evaluated at the annual and country scales. Individual genomes used in this study were sampled between 1983 and 2013.
Data exclusions	No data were excluded from the analyses.
Reproducibility	We did not conduct any experiments as part of this study, only analyses of preexisting data.
Randomization	Not applicable: we did not perform any experiments or interventions.
Blinding	Investigators were not blinded to any outcomes while performing data analyses.

Did the study involve field work? ☐ Yes ☒ No

Reporting for specific materials, systems and methods

We require information from authors about some types of materials, experimental systems and methods used in many studies. Here, indicate whether each material, system or method listed is relevant to your study. If you are not sure if a list item applies to your research, read the appropriate section before selecting a response.

Materials & experimental systems

n/a	Involved in the study
☒	☐ Antibodies
☒	☐ Eukaryotic cell lines
☒	☐ Palaeontology and archaeology
☒	☐ Animals and other organisms
☒	☐ Clinical data
☒	☐ Dual use research of concern
☒	☐ Plants

Methods

n/a	Involved in the study
☒	☐ ChIP-seq
☒	☐ Flow cytometry
☒	☐ MRI-based neuroimaging
